# Supplementary material for: A platform-agnostic deep reinforcement learning framework for effective Sim2Real transfer towards autonomous driving
Source: Commun Eng. 2024 Oct 17;3:147. doi: 10.1038/s44172-024-00292-3 (PMC11487131; doi:10.1038/s44172-024-00292-3)
Supplement: Supplementary file 2 — Supplementary Information [file 44172_2024_292_MOESM2_ESM.pdf]

Supplementary Information for A Platform-Agnostic Deep Reinforcement Learning Framework for Effective Sim2Real Transfer towards Autonomous Driving

SUPPLEMENTARY NOTE 1

To validate the robustness of the proposed Deep Reinforcement Learning (DRL) agent, additional tests are conducted using a different type of Duckiebot, DB19, which is equipped with a Raspberry Pi 3B. The DB19 has lower computational power and experiences high image transfer latency, which we approximate to be around 0.2 seconds in our network setup. Given the average speed of 0.4 m/s for the testing vehicles, this latency results in a forward movement of approximately 8 cm during the latency period. This delay is particularly critical when navigating sharp curves, challenging the robustness of the agent. As depicted in Supplementary Figure S1, when tested on DB19 with the proposed framework, it struggles with sharp turns but successfully navigates some curves, maintaining acceptable lane following behavior. Specifically, the proposed agent effectively guides the vehicle back to the right lane after encountering poor driving performance on curves due to latency issues. This demonstrates that the proposed framework can effectively mitigate the drawbacks imposed by the platform. It is worth noting that we also attempted to validate the E2E DRL and CNN DRL agents with DB19; however, they were unable to complete straight paths and a single curve, which is why their trajectories are not included in the plot.

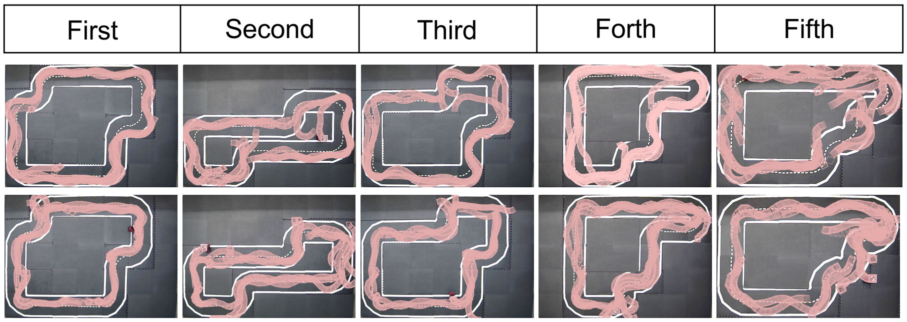

Supplementary Figure S1. Evaluation results of the proposed DRL agent on DB19 for lane following in real-world scenario.

SUPPLEMENTARY NOTE 2

This section discusses the quantitative evaluation results of three DB21 vehicles tested on a circular map, comparing the proposed DRL agent with the PID baseline. Supplementary Figure S2 presents the lane-following performance of two vehicles in a real-world scenario, while Supplementary Table S1 summarizes the performance metrics for both approaches.

SUPPLEMENTARY NOTE 3

In this section, we show parameters used during the training process. Supplementary Table S2 details the hyperparameters applied to the input states and reward function of the proposed DRL agent, while Supplementary Table S3 outlines the sub-reward functions. The hyperparameters

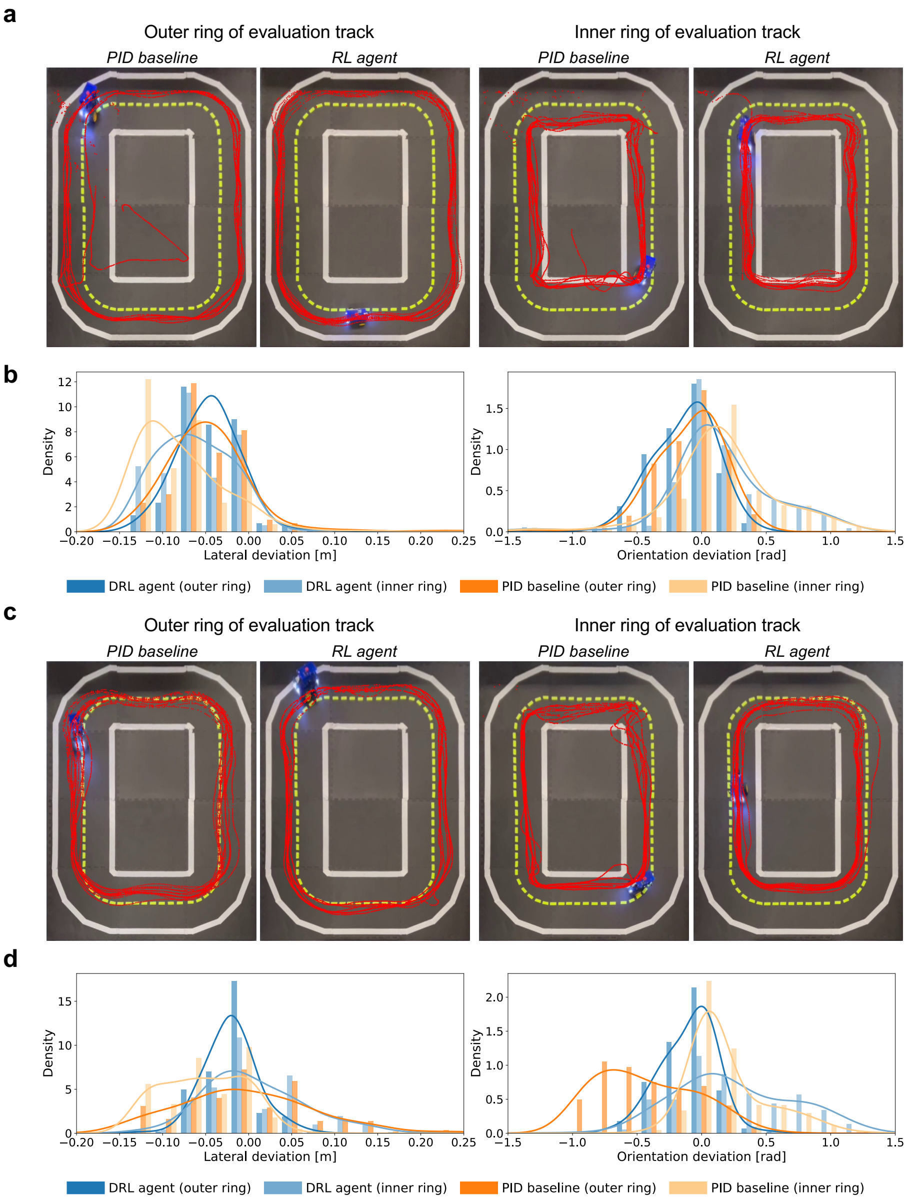

Supplementary Figure S2. Evaluation results of other two vehicles for lane following in real-world scenario. **a** and **c** are the illustration of the vehicle trajectories for different approaches i.e. DRL agent and PID baseline on the inner and outer ring of the real-world track for two vehicles respectively. **b** and **d** are the distribution histogram and kernel density estimate (KDE) plots of the lateral and orientation deviation for PID baseline and DRL agent during the real-world lane following evaluation for two vehicles respectively.

Supplementary Table S1. Evaluation results for real-world lane following task with different vehicles.

Vehicles	Driving direction	Control algorithm	Lateral deviation (δ_l)[1] Mean [m]	Lateral deviation (δ_l)[1] Stdv. [m]	Orientation deviation (δ_ϕ)[1] Mean [rad]	Orientation deviation (δ_ϕ)[1] Stdv. [rad]	Average Velocity [m/s]	Infraction[2]
Vehicle 1	Outer ring	PID baseline	-0.0301	0.0427	**-0.1208**	**0.2064**	0.4369	0
		DRL agent	**-0.0232**	**0.0423**	-0.2496	0.2808	**0.6999**	0
	Inner ring	PID baseline	**-0.0194**	0.0716	**0.0107**	0.4513	0.4371	8
		DRL agent	-0.0609	**0.0375**	0.1993	**0.4059**	**0.6004**	**0**
Vehicle 2	Outer ring	PID baseline	-0.0467	0.0509	**-0.0944**	0.2776	0.4399	2
		DRL agent	**-0.0466**	**0.0359**	-0.1292	**0.2401**	**0.7289**	**0**
	Inner ring	PID baseline	-0.0790	0.0512	0.1654	0.4050	0.4400	3
		DRL agent	**-0.0614**	**0.0446**	**0.1597**	**0.4039**	**0.6056**	**1**
Vehicle 3	Outer ring	PID baseline	**-0.0024**	0.0773	-0.4321	0.3821	0.4413	0
		DRL agent	-0.0209	**0.0312**	**-0.1033**	**0.2160**	**0.6423**	0
	Inner ring	PID baseline	-0.0528	0.0471	**0.1898**	**0.3031**	0.4427	2
		DRL agent	**0.0079**	**0.0618**	0.2461	0.4507	**0.5814**	**0**

[1] The lateral and orientation deviation in this table are not the exact value in the real world but the output from the perception module.
[2] Infraction in the real-world evaluation is counted whenever the agent drives off the road and needs to be relocated on the track by a human operator.
* The best performance for each track is highlighted in bold.

Supplementary Table S2. Hyperparameters for input state and reward function.

Parameter	Value
Lane width (d_{w})	0.23 m
Detection range(c_{d})	0.4 m
Reward factor (a)	0.001
Sensitivity parameter k_{r_c}	0.6

Supplementary Table S3. Sub-rewards function used for DRL agents.

Reward Term	Functions
Lane following reward	$R_{\mathrm{c},t} = a^{k_{r_c}\lvert e_{y,t}\rvert}$
Velocity bonus	$R_{\mathrm{v},t} = \frac{\lvert v_t - v_{\mathrm{desired}}\rvert}{v_{\mathrm{desired}}}$
Heading error reward	$R_{\theta,t} = \lvert\theta_t - \theta^*\rvert$

for training various DRL algorithms are provided in Supplementary Table S4, and the training performance of all agents is illustrated in Supplementary Figure S3.

SUPPLEMENTARY NOTE 4

In this section, we present the evaluation results of various DRL baselines alongside the proposed DRL agent. The trajectories are depicted in Supplementary Figure S4, and the detailed performance metrics are summarized in Supplementary Table S5.

SUPPLEMENTARY NOTE 5

In this section, we present the evaluation results of the trained DRL agent alongside other DRL baseline agents across various real-world environments, including scenarios with lane color changes (DC) and lane width variations (DW). The evaluation trajectories are depicted in Supplementary Figure S5, with detailed performance metrics provided in Supplementary Table S6.

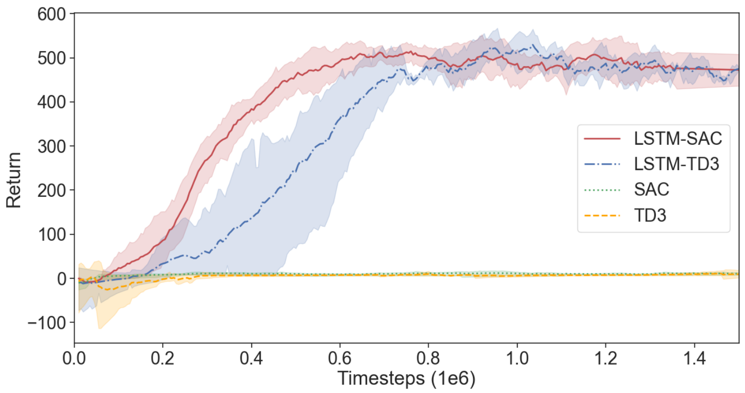

Supplementary Figure S3. Test return obtained by the different DRL agents during the training process, with shaded areas representing the standard deviations across 10 experiments.

Supplementary Table S4. List of hyperparameters used for DRL agents in the training process.

Hyperparameter	Value
Neural network structure[1]	$2 \times [256, \text{ReLU}]$
Loss function	MSELoss
Training batch size	100
Replay buffer size	10^5
Actor learning rate	10^{-4}
Critic learning rate	10^{-4}
Reward discount factor	0.99
Target update rate	0.005
Update start step	5000
Optimizer	Adam
Policy update delay[2]	2
History length for LSTM agent	2

[1] The activation functions of network outputs for Actor and Critic are *Tanh* and *Linear* respectively.
[2] Policy update delay is used in TD3 and LSTM-TD3.

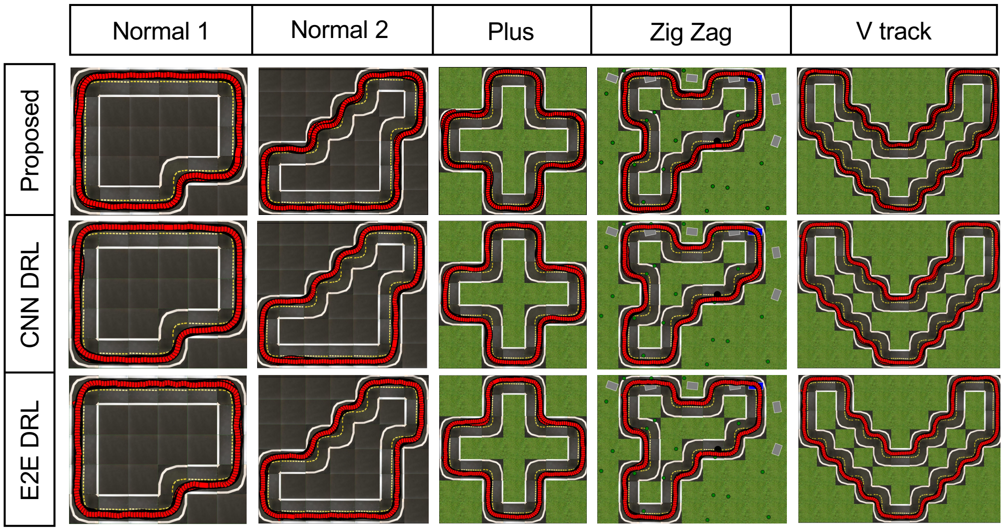

Supplementary Figure S4. Illustration of sampled vehicle trajectories for different Deep Reinforcement Learning (DRL) approaches within five different maps in Gym-Duckietown environment.

Supplementary Table S5. Evaluation results for different approaches within different tracks during the lane following evaluation in simulation.

Maps	Median metric over 100 episodes	Agents		
		Proposed	CNN DRL	E2E DRL
Normal 1	Final score	92.32	**92.50**	88.98
	Survival Time (T_s) [s][1]	60	60	60
	Traveled distance (d_t) [m]	35.80	**35.82**	32.67
	Lateral deviation (δ_l) [m·s]	1.13	**1.10**	1.37
	Orientation deviation (δ_ϕ) [rad·s]	4.71	**4.44**	4.65
	Major infractions (i_m) [s]	0.0	0.0	0.0
Normal 2	Final score	89.73	**90.13**	87.79
	Survival Time (T_s) [s]	60	60	60
	Traveled distance (d_t) [m]	34.50	**34.84**	32.53
	Lateral deviation (δ_l) [m·s]	1.68	1.64	**1.61**
	Orientation deviation (δ_ϕ) [rad·s]	6.18	**6.15**	6.26
	Major infractions (i_m) [s]	0.0	0.0	0.0
Plus track	Final score	89.43	**89.87**	88.55
	Survival Time (T_s) [s]	60	60	60
	Traveled distance (d_t) [m]	34.16	**34.26**	32.65
	Lateral deviation (δ_l) [m·s]	1.78	1.62	**1.42**
	Orientation deviation (δ_ϕ) [rad·s]	5.90	5.54	**5.37**
	Major infractions (i_m) [s]	0.0	**0.0**	0.0
Zig-Zag	Final score	88.62	**88.79**	87.72
	Survival Time (T_s) [s]	60	60	60
	Traveled distance (d_t) [m]	33.84	**34.03**	32.67
	Lateral deviation (δ_l) [m·s]	1.88	**1.86**	1.89
	Orientation deviation (δ_ϕ) [rad·s]	6.68	6.76	**6.12**
	Major infractions (i_m) [s]	0.0	0.0	0.0
V track	Final score	87.71	**87.72**	87.61
	Survival Time (T_s) [s]	60	60	60
	Traveled distance (d_t) [m]	33.43	**33.82**	32.51
	Lateral deviation (δ_l) [m·s]	1.97	1.98	**1.44**
	Orientation deviation (δ_ϕ) [rad·s]	7.50	8.24	**6.93**
	Major infractions (i_m) [s]	0.0	0.0	0.0

[1] The maximum evaluation time in one episode is 60s.
* The best performance for each track is highlighted in bold.

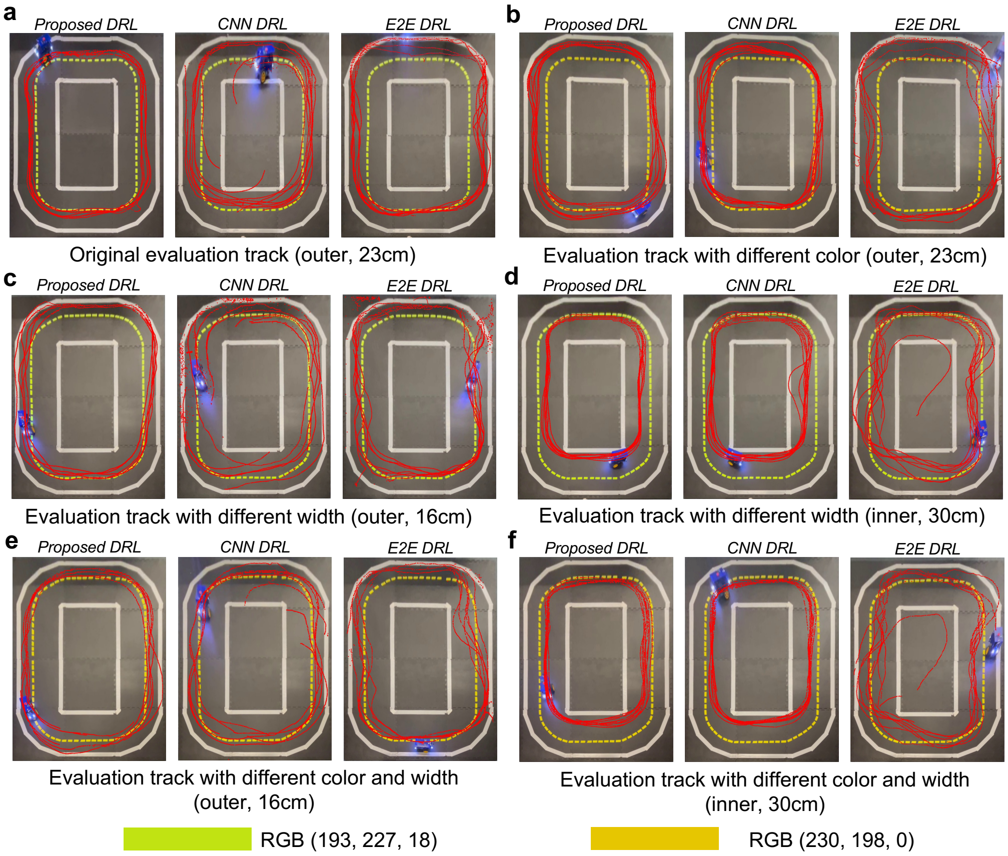

Supplementary Figure S5. Evaluation results of the proposed Deep Reinforcement Learning (DRL) agent and two other machine learning based baselines for lane following in real-world scenario. **a**∼**f** are the illustration of the vehicle trajectories for different approaches in different real-world setups.

Supplementary Table S6. Number of infractions during the real-world evaluation for three DRL agents in different setups.

Real-world setups	Proposed DRL	CNN DRL	E2E DRL
Original	**0**	3	1
DC[1]	0	0	7
DW[2](inner, 30cm)	0	0	1
DW (outer, 16cm)	**0**	8	7
DC, DW (inner, 30cm)	0	0	1
DC, DW (outer, 16cm)	**0**	3	7

[1] DC: Different colors are used for evaluation compared with the original lane color color.
[2] DW: Different lane widths are applied for evaluation, with the inner ring with 30cm of lane width and the outer ring with 16cm lane width.
* The best performance for each track is highlighted in bold.
